# Restoration of Transgelin 2 Expression Reverses Immune Escape in Ovarian Cancer: A Dawn for Immunotherapy

**DOI:** 10.1002/mco2.70164

**Published:** 2025-03-30

**Authors:** Zhiqiang Wang, Ge Lou, Mingzhu Yin

**Affiliations:** ^1^ Department of Gynecology Oncology Harbin Medical University Cancer Hospital Harbin China; ^2^ Clinical Research Center, Medical Pathology Center, Cancer Early Detection and Treatment Center, and Translational Medicine Research Center Chongqing University Three Gorges Hospital, Chongqing University Chongqing China; ^3^ Department of Dermatology, Hunan Engineering Research Center of Skin Health and Disease, Hunan Key Laboratory of Skin Cancer and Psoriasis, Xiangya Hospital Central South University Changsha China

1

In a seminal study published in *Nature*, Hwang et al. recently revealed for the first time that Transgelin 2 (TAGLN2) regulates lipid metabolism and antitumor function through interaction with fatty acid binding protein 5 (FABP5) in CD8+ T cells [1]. The mechanism by which TAGLN2 is inhibited by endoplasmic reticulum (ER) stress in the microenvironment of ovarian cancer (OC) leading to T‐cell dysfunction was clarified, providing a new target and a potential strategy for improving immunotherapy for solid tumors.

Lipid metabolism plays an indispensable role in the activation, proliferation, and execution of effector functions in T cells [2]. As a critical regulator of lipid metabolism, FABP5 efficiently coordinates lipid uptake and transport, providing sufficient energy substrates for mitochondrial respiration in T cells, thereby ensuring their optimal bioenergetic status during antitumor immunity [3, 4]. However, significant gaps remain in understanding the regulatory mechanisms of FABP5 in T‐cell lipid metabolism, particularly within tumor‐related contexts. Metastatic OC, a refractory immunosuppressive tumor resistant to multiple therapeutic approaches—including T cell‐based immunotherapies, exerts a microenvironment that drives functional impairment in tumor‐infiltrating T cells [5]. Consequently, elucidating the molecular mechanisms underlying immune evasion in OC and identifying potential therapeutic targets capable of reversing T cell dysfunction is paramount for enhancing the efficacy of OC immunotherapy and improving patient prognosis.

The exact way tumor microenvironment (TME) inhibits T cell lipid metabolism is unclear. Hwang et al. demonstrated that TAGLN2 expression in CD8+ T cells was significantly downregulated in both ascites and solid tumors of OC patients. In addition, CD8+ T cell surface FABP5 expression was decreased in ascites from OC patients compared to peripheral CD8+ T cells from cancer‐free individuals, although total intracellular FABP5 levels were comparable. These results suggest that the OC microenvironment may affect the lipid metabolism and function of intratumoral T cells by repressing TAGLN2 expression. Further experiments showed that the interaction of TAGLN2 with FABP5 is critical for the localization and function of FABP5 on the surface of activated CD8+ T cells. This allows the cells to efficiently take up extracellular fatty acids to fuel mitochondrial respiration, thereby maintaining the energy requirements of CD8+ T cells. These results emphasize the central role of TAGLN2 in T‐cell lipid metabolism, particularly in promoting FABP5‐mediated lipid uptake.

What molecular pathways link TME stress signals to TAGLN2 suppression?​ The authors discovered that the ER stress response inhibits TAGLN2 expression through the IRE1α‐XBP1s signaling pathway. The active form of XBP1s can bind directly to the promoter region of the *TAGLN2* gene and inhibit its transcription. Overexpression of TAGLN2 rescued bioenergetic deficits in ER‐stressed CD8+ T cells, enhanced lipid uptake, mitochondrial respiration, and expression of effector cytokines, and promoted extracellular fatty acid utilization under glucose‐limited conditions (Figure 1).

**FIGURE 1:**
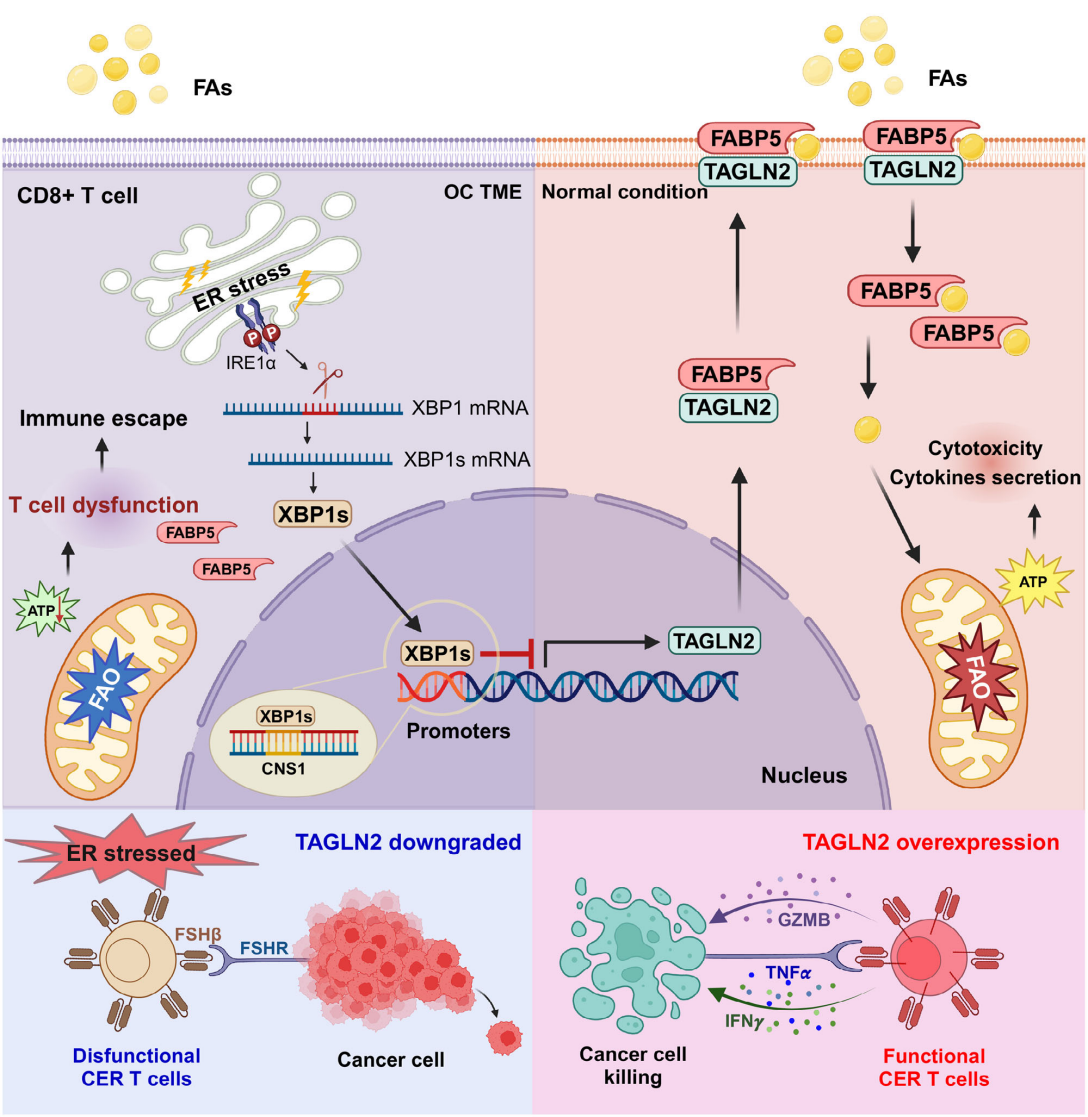
ER stress‐induced XBP1s silences TAGLN2 and thereby inhibits CD8⁺ T cell lipid metabolism to induce immune escape in ovarian cancer. Under normal conditions, TAGLN2 interacts with FABP5 to promote cell surface localization and ensure stable T‐cell lipid uptake, fatty acid oxidation (FAO), mitochondrial respiration, and effector functions. However, in ovarian cancer TME, endoplasmic reticulum (ER) stress is induced, leading to the activation of XBP1s, which binds to the promoter region of the *TAGLN2* gene and represses TAGLN2 expression. As a result, the surface localization of FABP5 is disrupted, impairing lipid uptake and metabolism in CD8+ T cells and facilitating tumor immune escape. Through overexpression of TAGLN2, CER T cells circumvented ER stress‐mediated functional suppression in the TME and demonstrated significantly enhanced effector functions, persistence, and antitumor activity. TME, tumor immune microenvironment; FAs, fatty acids.

In the context of OC, T‐cell immunotherapies, such as chimeric antigen receptor (CAR) T cells, often demonstrate limited efficacy against solid malignancies. Could engineering TAGLN2 expression overcome the limitations of current T‐cell therapies?​ Overexpression of TAGLN2 in chimeric endocrine receptor (CER) T cells, which leverage the two subunits of follicle‐stimulating hormone (FSH) to specifically target and eliminate FSH‐receptor positive (FSHR+) ovarian tumors, increased their therapeutic efficacy. These cells exhibited enhanced persistence, expanded central memory populations, and superior tumor control in OC mouse models, independent of PD‐1 blockade. This highlights TAGLN2 as a metabolic checkpoint and a tunable node to arm T cells against the immunosuppressive TME.

This study, for the first time, unambiguously defines the crucial role of TAGLN2 in T‐cell lipid metabolism. It discovers that TAGLN2 interacts with FABP5 to maintain T cell lipid uptake and function, thus filling a significant gap in understanding the regulatory mechanisms of T cell immunometabolism. The research reveals that the OC microenvironment inhibits TAGLN2 expression through the ER stress‐IRE1α‐XBP1s pathway, leading to T‐cell dysfunction and immune escape. This finding provides a novel perspective for understanding tumor immune escape mechanisms. Moreover, it is demonstrated that maintaining TAGLN2 expression can improve the efficacy of immunotherapies such as CAR‐T cell therapy, offering potential new targets and strategies for enhancing the effectiveness of immunotherapy against solid tumors.

Of course, this study has some limitations. The researchers mainly relied on the mouse model to investigate the function of TAGLN2 in T cells and related mechanisms. Although the mouse model plays an essential role in the study, it cannot be ignored that the differences in physiology and immune system between the mouse model and the human body may affect the direct application of the research results to human clinical treatment. The scope of the study focused on OC. However, given the unique microenvironmental characteristics, molecular pathogenesis, and immune escape strategies of different tumors, the expression patterns, functional roles, and regulatory mechanisms of TAGLN2 in other tumors may differ. Although CAR‐T cells with overexpressed TAGLN2 have demonstrated promising therapeutic effects in experiments, their clinical application's safety and potential side effects still necessitate further evaluation. At the same time, the complex interactions between TAGLN2 and other intracellular signaling pathways have yet to be explored in depth, which may limit the comprehensive understanding of its regulatory mechanism and thus affect the optimization of therapeutic strategies.

In the future, the study should be expanded to more common solid tumors, and the characteristics of TAGLN2 in different tumor microenvironments should be investigated in depth to clarify its universality and specificity. We should comprehensively analyze the interaction network between TAGLN2 and many intracellular signaling pathways, such as the T‐cell receptor signaling pathway, costimulatory signaling pathway, and other metabolic‐related pathways, and explore more potential regulatory nodes. ER stress is an important factor to consider in the development and application of CAR T cell therapy. To vigorously carry out clinical trials of TAGLN2‐based combination therapy and optimize the parameters of the combination protocol. Explore the potential of TAGLN2 as a biomarker to develop personalized and precise treatment strategies. At the same time, we will further explore the mystery of TAGLN2 in the remodeling of the tumor microenvironment to open up new paths for developing novel tumor treatment strategies and enhancing immunotherapy efficacy.

## Author Contributions

All authors were involved in the writing of the manuscript. Zhiqiang Wang and Mingzhu Yin initiated the conception and outline. Zhiqiang Wang and Ge Lou organized and processed the figure. All authors have read and approved the final manuscript.

## Ethics Approval

The authors have nothing to report.

## Conflicts of Interest

The authors declare no conflicts of interest.

## Data Availability

The authors have nothing to report.
